# Serial coronary computed tomography angiography-verified coronary plaque progression: comparison of stented patients with or without diabetes

**DOI:** 10.1186/s12933-019-0924-z

**Published:** 2019-09-24

**Authors:** Rui Shi, Ke Shi, Zhi-gang Yang, Ying-kun Guo, Kai-yue Diao, Yue Gao, Yi Zhang, Shan Huang

**Affiliations:** 1 Department of Radiology, West China Hospital, Sichuan University, 37# Guo Xue Xiang, Chengdu, 610041 Sichuan China; 2 Department of Radiology, Key Laboratory of Birth Defects and Related Diseases of Women and Children of Ministry of Education, West China Second University Hospital, Sichuan University, Chengdu, China

**Keywords:** Type 2 diabetes mellitus, Coronary artery computed tomography, Percutaneous coronary intervention, Coronary plaques

## Abstract

**Background:**

Patients with Diabetes mellitus (DM) are susceptible to coronary artery disease (CAD). However, the impact of DM on plaque progression in the non-stented segments of stent-implanted patients has been rarely reported. This study aimed to evaluate the impact of DM on the prevalence, characteristics and severity of coronary computed tomography angiography (CCTA) verified plaque progression in stented patients. A comparison between diabetic and non-diabetic patients was performed.

**Methods:**

A total of 98 patients who underwent clinically indicated serial CCTAs arranged within 1 month before and at least 6 months after percutaneous coronary intervention (PCI) were consecutively included. All the subjects were categorized into diabetic group (n = 36) and non-diabetic groups (n = 62). Coronary stenosis extent scores, segment involvement scores (SIS), segment stenosis scores (SSS) at baseline and follow-up CCTA were quantitatively assessed. The prevalence, characteristics and severity of plaque progression was evaluated blindly to the clinical data and compared between the groups.

**Results:**

During the median 1.5 year follow up, a larger number of patients (72.2% vs 40.3%, P = 0.002), more non-stented vessels (55.7% vs 23.2%, P < 0.001) and non-stented segments (10.3% vs 4.4%, P < 0.001) showed plaque progression in DM group, compared to non-DM controls. More progressive lesions in DM patients were found to be non-calcified plaques (31.1% vs 12.8%, P = 0.014) or non-stenotic segments (6.6% vs 3.0%, p = 0.005) and were more widely distributed on left main artery (24.2% vs 5.2%, p = 0.007), the right coronary artery (50% vs 21.1%, P = 0.028) and the proximal left anterior artery (33.3% vs 5.1%, P = 0.009) compared to non-DM patients. In addition, DM patients possessed higher numbers of progressive segments per patient, ΔSIS and ΔSSS compared with non-DM individuals (P < 0.001, P = 0.029 and P < 0.001 respectively). A larger number of patients with at least two progressive lesions were found in the DM group (P = 0.006). Multivariate logistic regression analysis demonstrated that DM (OR: 4.81; 95% CI 1.64–14.07, P = 0.004) was independently associated with plaque progression.

**Conclusions:**

DM is closely associated with the prevalence and severity of CCTA verified CAD progression. These findings suggest that physicians should pay attention to non-stent segments and the management of non-stent segment plaque progression, particularly to DM patients.

## Background

Diabetes Mellitus (DM) is a chronic disease that endangers human health, causing a severe socioeconomic burden. The International Diabetes Federation’s global estimates suggest that ~ 422 million individuals suffer from DM worldwide, and this number is projected to increase to 642 million by 2040 [1]. Cardiovascular involvement increases the risk of adverse events in DM patients [2, 3]. Serial CCTAs demonstrated that DM patients have a larger numbers of atherosclerotic plaques compared to non-DM patients at an interval of median 3.2 years [4]. The role of DM in CAD progression is now universally accepted [5–7].

The susceptibility of DM patients to a severe plaques and PCI is well-known [8, 9]. DM patients are more likely to develop in-stent restenosis, leading to a poorer clinical outcomes and higher target lesion revascularization rates after PCI [10, 11]. The advent of drug eluting stents (DES) has decreased restenosis rates [12]. Poor outcomes in CAD patients not only originate from in-stent restenosis, but also non-treated segments [13]. The role of untreated and non-stenotic segments during CAD progression and their relationship to secondary adverse cardiovascular events (MACE) remain undefined.

Assessments of atheromatous plaque progression between elective PCI treated patients with or without DM are sparse. The aim of this study was to compare the prevalence and severity of plaque progression in stented subjects with and without diabetes and to compare the between-group differences in the characteristics and distribution of these progressive plaques through serial CCTAs.

## Methods

### Study population

From December 2015 to October 2018, 106 patients who underwent serial CCTA examinations in our hospital were consecutively included. Baseline CCTAs were performed for angina, suspected angina, abnormal ECGs, and the preoperative evaluation or screening of CAD in the population with multiple risk factors. Follow-up CCTA were performed for postoperative checks. Inclusion criteria were: (i) elective percutaneous coronary intervention (PCI) performed in our hospital without prior known CAD; (ii) clinically indicated serial CCTAs arranged 1 month before and at least 6 months after PCI. Exclusion criteria were as follows: (i) repeated revascularization; and (ii) scans with significant artifacts or poor image quality. A total of 98 patients were finally included. Eight patients were excluded for poor image quality (n = 3) or shorter inter-scan periods (n = 5). All subjects were categorized into 2 groups according to DM history (DM and non-DM groups). DM was defined in accordance with the American Diabetes Association (ADA) diagnosis [14]. Clinical variables, CAD risk factors and statin use within the groups were obtained through patient questionnaires and medical records.

### CCTA scanning protocols

CCTAs indications, data acquisition and image post-processing were performed in accordance with the Society of Cardiovascular Computed Tomography guidelines [15]. CCTA was performed using a Siemens DSCT scanner (SOMATOM Definition, Siemens Medical Solutions, Forchheim, Germany). Beta blockers were not administered for heart rate reduction. The scanning scope was from the tracheal bifurcation to 20 mm below the inferior cardiac apex. A 70–90-mL (dependent on the body mass index) bolus of iodinated contrast agent (iopamidol, 370 mg of iodine/mL; BraccoSine Pharmaceutical Corp. Ltd, Shanghai, China) was injected into the antecubital vein at a flow rate of 5 mL/s. Next, a 20-mL saline chaser was injected at the same rate. Scan parameters were tube voltage 100–120 kV (adapted to body mass index); tube current, 220 mAs; collimation, 64 × 0.6 mm; rotation time, 0.33 s and pitch, 0.2–0.5 (adapted to the heart rate). Retrospective electrocardiographic gating was used to eliminate cardiac motion artefacts. Initial datasets were immediately reconstructed upon completion of the scan and images of optimal quality were transferred to a post-processing workstation (Syngo-Imaging, Siemens Medical Solution Systems, Forchheim, Germany) for image analysis. Sinogram Affirmed Iterative Reconstruction (SAFIRE) was used when plaques were highly calcified, to reduce image noise and optimize image quality. Coronary artery plaques were evaluated through maximum intensity projections, multiplanar reconstructions, curvature plane reconstructions and volume reconstructions.

### Serial CCTAs analysis

**Fig. 1:**
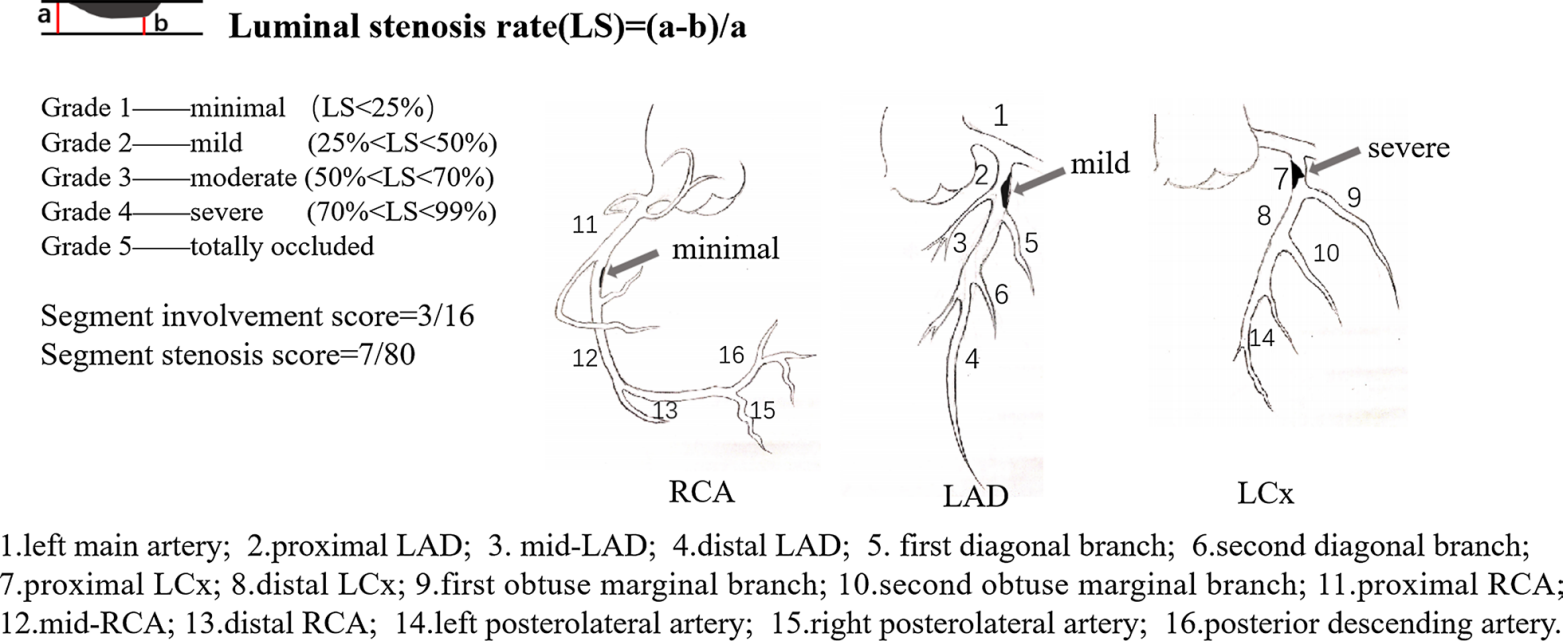
Schematic diagram of coronary artery plaque score in coronary artery tree model. In this example, plaques distribute on proximal right coronary artery, proximal left anterior descending and proximal left circumflex respectively. Segment stenosis score was calculated by summation of minimal plaque in the proximal right coronary artery (scored 1), severe plaque in the proximal left anterior descending artery (scored 4) and mild plaque (scored 2) in the proximal left circumflex. Thus, the segment stenosis score is 7 out of a possible 80. Segment involvement score was calculated by summation of the absolute number of coronary segments exhibiting plaque. The segment involvement score in this example is 3 out of a possible 16

For longitudinal comparisons of the CCTAs, segments and vessels were identified by landmarks including bifurcations carina and side branches at follow-up. Segments and vessels with stents on follow-up CCTA (CCTA2) images were excluded from the analyses. Finally, an equal number of segments and vessels were assessed at baseline and follow-up. For segment-wise analysis, coronary artery trees were divided into 16 separate segments based on a modified AHA classification (Fig. 1) [16]. The extent of stenosis in the individual segments was qualitatively analyzed and graded by two professional cardiologists who were masked to the clinical results and group identities, using a 5-point scale based on CAD-RADS [17]: Grade 0: absence of plaques; Grade 1-minimal (< 25% luminal stenosis); Grade 2-mild (25–50% luminal stenosis); Grade 3-moderate (50–70% luminal stenosis); Grade 4-severe (70–99% luminal stenosis); Grade 5-totally occluded. Any discrepancies in the interpretations of the two observers were resolved by consensus.

Segment involvement scores (SIS) reflecting the extent of stenosis were calculated as the total number of coronary artery segments with plaques (minimum 0, maximum 16). Segment stenosis scores (SSS) reflecting the severity of stenosis were calculated as the summation of the extent scores of all 16 individual segments (scale: 0 to 80). These two variables were calculated based on the final results of the segmentation assessments of the two cardiologists. SSS (ΔSSS) and SIS changes (ΔSIS), as variables to evaluate the severity of atheromatous plaque progression, were defined as SSS and SIS at CCTA2 minus that of CCTA1. Plaques were visually classified as calcified when containing a calcified composition, or non-calcified plaques containing partial or no-calcification.

**Fig. 2:**
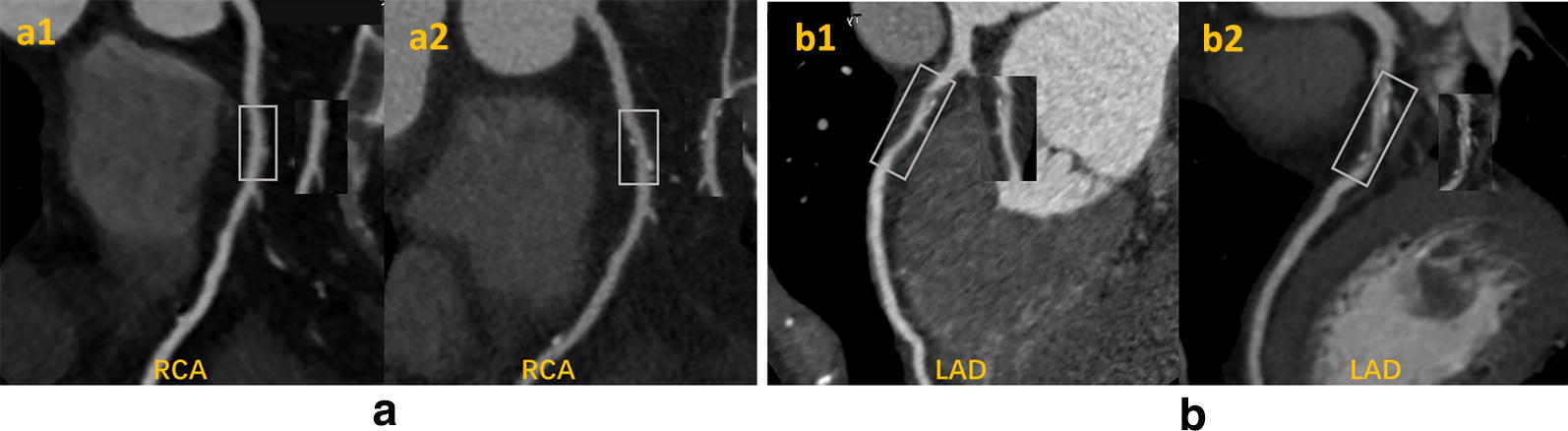
Representative serial CCTAs images. **a** A serial CCTAs of a non-diabetic patient. At the baseline CCTA (a1) (performed on April, 2014), no plaque was found on the proximal right coronary artery (pRCA). At the second CCTA (a2) (performed on July, 2018), a newly developed calcific plaque was noticed on the pRCA with minimal stenosis. **b** A serial CCTAs of a diabetic patient. At the baseline CCTA (b1) performed on February, 2013, a mixed plaque with mild stenosis was found on the proximal left anterior descending artery (pLAD). At the second CCTA (b2) performed on March, 2017, the mixed plaque on the pLAD has progressed into severe stenosis

The prevalence of atheromatous plaque progression was assessed at per-patient, per-vessel, and per-segment levels. For patient analysis, CAD progression was defined as the increase in extent scores of any coronary segment in the coronary artery tree. For per-vessel analysis, non-stent vessels with progressive segments on CCTA2 were defined as progressive vessels. On the per-segment level, coronary plaque progression was defined as an increased extent score of individual segments. Figure 2 shows representative CCTA images for plaque progression. The severity of plaque progression was assessed mainly by ΔSSS, ΔSIS, and the number of progressive vessels and segments.

### Statistical analysis

Statistical analysis was performed using SPSS software (version 25.0). Baseline clinical and imaging data were stratified based on DM status. Categorical variables were expressed as the number (%) and compared using Chi square test or fisher’s exact test (if the expected cell value was ≤ 5). Continuous variables were expressed as the mean ± standard deviation for normally distributed data or the Median (interquartile range) for non-normally distributed data. Normally distributed continuous variables such as age and BMI were compared using an unpaired Students *t* test. For non-normally distributed variables such as SSS, SIS, ΔSIS and ΔSSS, non-parametric tests were used. To compare baseline CTA data and the changes over time between the two groups, Mann–Whitney U tests were employed. The correlation between plaque progression and clinical variables were analyzed using logistic regression analysis. Variables with P-values ≤ 0.1 in univariate analysis and recognized cardiovascular risk factors were entered en-bloc into the multivariable model. Two-tailed P-values < 0.05 were considered statistically significant.

## Results

### Study population

A total of 256 non-stent vessels (DM vs non-DM: 88 vs 168) with 1355 non-stented segments (DM vs non-DM: 485 vs 870) in 98 stented patients (DM vs non-DM: 32 vs 36) were evaluated. The mean age of the participants was 69.9 ± 11.0 years, and 83.7% were male. The clinical characteristics of DM and non-DM patients are shown in Table 1. Higher BMIs were observed in DM individuals (P = 0.038), whilst no significant discrepancies regarding age, sex, high-risk factors and inter-scan periods between the two groups. Amongst the stents, there were no significant differences in distribution, burden, or stents involving segments and vessels between DM and non-DM groups (P > 0.05). The use of statins, clopidogrel/ticlopidine and other prescriptions at discharge did not significantly differ between the two study groups.

**Table 1:** Baseline characteristics of participates in the two groups

Characteristics	DM (n = 36)	Non-DM (n = 62)	P-value
Age, y	69.6 ± 10.3	70.0 ± 11.5	0.389
Male	30 (83.3)	52 (83.9)	0.945
BMI, kg/m^2^	24.4 ± 3.8	23.1 ± 2.7	0.038
Systolic BP, mmHg	141.9 ± 36.6	132.8 ± 19.8	0.287
Diastolic BP, mmHg	81.6 ± 12.2	79.2 ± 8.3	0.447
Hypertension	26 (72.2)	36 (58.1)	0.161
Dyslipidemia	9 (25)	10 (16.1)	0.284
Current smoking	11 (30.6)	21 (33.9)	0.736
CVD	8 (22.2)	7 (11.3)	0.147
CAD family history	4 (11.1)	5 (8.1)	0.888
Chest pain	22 (61.1)	42 (67.7)	0.506
Anti-diabetic treatment			
Insulin	9 (25.0)	–	–
Metformin	13 (36.1)	–	–
Sulfonylurea	5 (13.9)	–	–
α-Glucosidase inhibitor	14 (38.9)	–	–
Non-drug	2 (5.5)	–	–
Statins at discharge	29 (80.6)	47 (75.8)	0.120
Clopidogrel/ticlopidine at discharge	36 (100)	62 (100)	–
Inter-scan period, y	1.8 (1–3.5)	1 (0.5–2.5)	0.171
No. of stented vessel/patients	1 (1–2)	1 (1–2)	0.235
No. of stented lesions/patients	2 (1–3.75)	2 (1–2.25)	0.235
Number of stented segments			
1	15 (41.7)	30 (48.4)	0.520
2	8 (22.2)	17 (27.4)	0.569
3	4 (11.1)	6 (9.7)	1
≥ 4	9 (25)	9 (14.5)	0.196
Number of stented vessels			
1	22 (61.6)	44 (71.0)	0.316
2	9 (25)	15 (24.2)	0.929
≥ 3	5 (13.9)	3 (4.8)	0.139
Stent site			
LM	3 (8.3)	4 (6.5)	0.705
LAD	25 (69.4)	35 (56.5)	0.203
RCA	18 (50)	24 (38.7)	0.276
LCx	10 (27.8)	17 (27.4)	0.969

Values are expressed as mean ± SD, median (interquartile range) or n (%)

*BP* blood pressure, *CVD* cerebrovascular disease, *BMI* body mass index, *CAD* coronary artery disease, *LM* left main, *LAD* left anterior descending, *RCA* right coronary artery, *LCx* left circumflex

### Prevalence and characteristics of progressive atheromatous plaques in DM and non-DM patients

The prevalence and characteristics of progressive atheromatous plaques are shown in Table 2. During the median 1.5 years follow-up, 26 (72.2%) DM patients and 25 (40.3%) non-DM patients developed progressive atheromatous plaques (P = 0.002). A larger number of atheromatous plaques progressions were observed in the non-stented vessels (55.7% vs 23.2%, P < 0.001) and non-stented segments (10.3% vs 4.4%, P < 0.001) of DM patients compared to non-DM patients.

**Table 2:** Atheromatous plaque progression and characteristics assessment in DM and non- DM patients

	Total	Progressor			
	DM/non-DM (O1/O2)	DM	Non-DM	P	OR (95% CI)
Patients	36/62	26 (72.2)	25 (40.3)	0.002	3.85 (1.58–9.36)
Vessels/segments					
LM	33/58	8 (24.2)	3 (5.2)	0.007	5.86 (1.43–23.99)
LAD	11/25	6 (54.5)	10 (40)	0.483	1.80 (0.43–7.53)
LAD1	18/39	6 (33.3)	2 (5.1)	0.009	9.25 (1.64–52.06)
LAD2	16/32	2 (12.5)	7 (21.9)	0.697	0.51 (0.09–2.80)
LAD3	28/54	2 (7.1)	4 (7.4)	1	0.96 (0.16–5.60)
D1	36/62	4 (11.1)	1 (1.6)	0.059	7.63 (0.82–71.10)
D2	36/62	2 (5.6)	0 (0)	0.133	–
RCA	18/38	9 (50)	8 (21.1)	0.028	3.75 (1.12–12.56)
RCA1	22/48	5 (22.7)	3 (6.3)	0.098	4.41 (0.95–20.50)
RCA2	25/46	4 (16.0)	2 (4.3)	0.175	4.19 (0.71–24.73)
RCA3	32/57	6 (18.8)	7 (12.3)	0.533	1.65 (0.50–5.41)
r-PDA	36/62	0	0	–	–
r-PLB	36/61	1 (2.8)	1 (1.6)	1	1.71 (0.10–28.28)
LCx	26/44	7 (26.9)	4 (9.1)	0.085	3.68 (0.96–14.13)
LCx1	29/54	4 (13.8)	5 (9.3)	0.713	1.57 (0.39–6.36)
LCx2	30/49	6 (20)	2 (4.1)	0.048	5.89 (1.10–31.34)
OM1	36/62	0	1 (1.6)	1	–
OM2	36/62	0	0	–	–
L-PDA	36/62	0	0	–	–
Plaque type					
Calcified plaque	54/106	10 (18.5)	11 (10.4)	0.149	1.96 (0.78–4.97)
Non-calcified plaque	45/78	14 (31.1)	10 (12.8)	0.014	3.07 (1.23–7.68)
Original extent score					
0	362/709	24 (6.6)	21 (3.0)	0.005	2.33 (1.28–4.24)
1	17/27	8 (47.1)	5 (18.5)	0.043	3.91 (1.00–15.24)
2	62/117	9 (14.5)	11 (9.4)	0.301	1.64 (0.64–4.19)
3	15/23	6 (40)	3 (13.0)	0.128	4.44 (0.90–21.87)
4	4/10	1 (25.0)	0 (0)	0.286	0.23 (0.09–0.62)

Values are expressed as n or n (%)

*O1* the corresponding number of observations in the DM group, *O2* the corresponding number of observations in the non-DM group, *LM* left main, *LAD* left anterior descending, *RCA* right coronary artery, *LCx* left circumflex, *PDA* posterior descending artery, *PLB* posterolateral artery, *D1* first diagonal branch, *D2* second diagonal branch, *OM1* first obtuse marginal branch, *OM2* second obtuse marginal branch

The larger number of progressive lesions in DM patients distributed on the main left artery (LM) (24.2% vs 5.2%, P = 0.007) and right coronary artery (RCA) (50% vs 21.1%, P = 0.028) compared to non-DM patients. For segment-wise analysis, proximal left anterior descending (LADs) of DM patients were more prone to plaque progression than those of non-DM patients (33.3% vs 5.1%, P = 0.009). A larger number of calcified plaques and original non-stenotic segments with plaque progression were observed in DM compared to non-DM patients (P = 0.014 and P = 0.005, respectively).

### Severity and extent of atheromatous plaque progression in DM and non-DM patients

Approximately 1.0 (interquartile range (IQR): 0–2.0) segment per DM patient and 0 (IQR: 0–1) segments per non-DM patient were found to possess plaques progression (P = 0.001). A larger number of DM patients had at least two progressive segments compared to non-DM patients (P = 0.006). No significant between-group differences were observed in patients with single progressive segments (P > 0.05). The number of progressive vessels between the two groups also did not differ (P > 0.05).

**Fig. 3:**
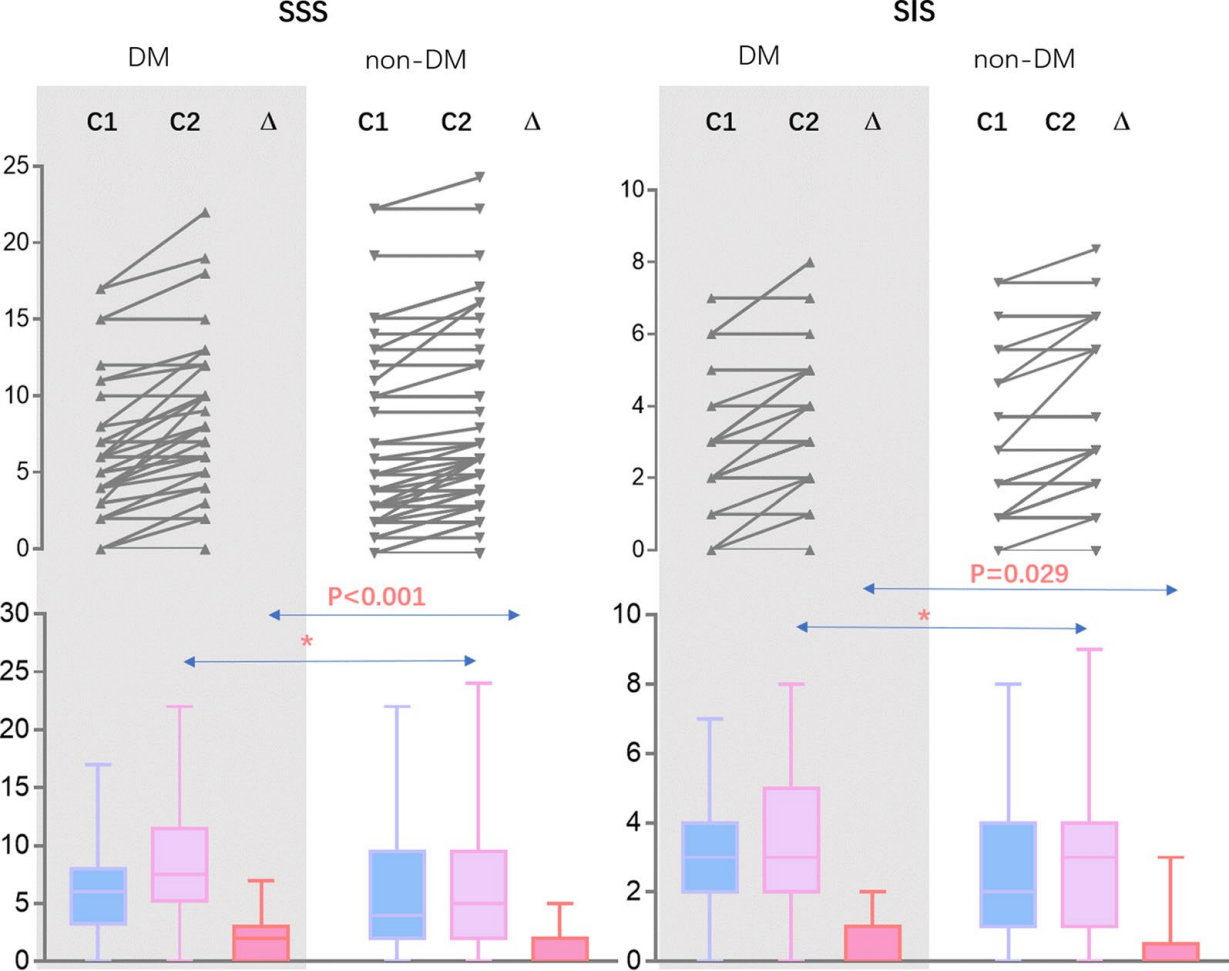
Assessment of plaque burden and extent in DM and non-DM patients. *No statistical differences. *SSS* segment stenosis score, *SIS* segment involvement score, *DM* diabetes mellitus, *non-DM* non-diabetic mellitus, *C1* baseline CCTA (CCTA1), *C2* follow-up CCTA (CCTA2)

The segment stenosis score (SSS) relatively increased from 6 (IQR: 3.25–8) to 7.5 (IQR: 5.25–11.5) in DM patients, and from 4 (IQR: 2–10) to 5 (IQR: 2–10) in non-DM patients (P < 0.001 for between-group differences in change). SIS scores increased from 3 (IQR: 2–4) to 3 (IQR: 2–5) in DM patients and from 2 (IQR: 1–4) to 3 (IQR: 1–4) in non-DM patients (P = 0.029 for between-group differences in change, Fig. 3, Table 3).

**Table 3:** Severity of plaque progression assessment in DM and non-DM patients

	DM (n = 36)	Non-DM (n = 62)	P	OR (95% CI)
Number of progressive segments/patient	1.0 (0–2.0)	0 (0–1)	0.001	–
Number of progressive vessels/patients	0.5 (0–1)	0 (0–1)	0.055	–
Number of progressive segments				
0	10 (27.8)	37 (59.7)	0.002	0.32 (0.13–0.77)
1	12 (33.3)	16 (25.8)	0.427	1.44 (0.59–3.52)
≥ 2	14 (19.4)	9 (14.5)	0.006	3.75 (1.42–9.92)
Number of progressive vessels				
0	18 (50)	41 (66.1)	0.116	0.51 (0.22–1.19)
1	11 (30.6)	18 (29.0)	0.873	1.08 (0.44–2.64)
2	5 (13.9)	2 (3.2)	0.096	4.84 (0.89–26.39)
3	2 (5.6)	1 (1.6)	0.552	3.59 (0.31–41.34)
ΔSIS	0 (0–1)	0 (0–0.25)	0.029	–
ΔSSS	2 (0–3)	0 (0–2)	< 0.001	–

Values are expressed as median (interquartile range) or n (%)

*SSS* segment stenosis score, *SIS* segment involvement score change, *ΔSIS* segment involvement score, *ΔSSS* segment stenosis score changes

### Univariate and multivariate analysis

The results of univariate and multivariate regression analyses are shown in Table 4. In the univariate analysis, the inter-scan period, chest pain at baseline, cardiovascular disease (CVD) and DM were significantly associated with plaque progression. Multivariate regression analysis when adjusted for confounding factors demonstrated that DM (OR: 4.81; 95% CI 1.64–14.07, P = 0.004) and chest pain at baseline (OR: 3.55; 95% CI 1.22–10.32, P = 0.020) were independently associated with plaque progression.

**Table 4:** Independent predictors of atherosclerosis progression

	Univariate		Multivariate	
	OR (95% CI)	P	OR (95% CI)	P
Sex	1.10 (0.38–3.22)	0.858	0.80 (0.19–3.47)	0.769
Age	1.00 (0.97–1.05)	0.674	0.99 (0.95–1.04)	0.748
BMI	0.96 (0.85–1.09)	0.564	0.89 (0.75–1.05)	0.172
Hypertension	1.62 (0.71–3.71)	0.253	1.25 (0.45–3.52)	0.669
Hyperlipidemia	1.34 (0.49–3.69)	0.57	0.97 (0.30–3.16)	0.960
Current smoking	1.07 (0.46–2.49)	0.881	1.14 (0.39–3.31)	0.811
DM	3.85 (1.59–9.36)	0.003	4.81 (1.64–14.07)	0.004
Chest pain	2.86 (1.21–6.78)	0.017	3.55 (1.22–10.32)	0.020
CVD	4.51 (1.19–17.18)	0.027	4.57 (1.02–20.42)	0.047
Interscan-period	1.47 (1.11–1.95)	0.007	1.33 (0.95–1.86)	0.094

*DM* diabetic mellitus, *CVD* cerebrovascular disease, *BMI* body mass index

## Discussion

The main findings of this study were that (1) compared to non-DM patients, diabetic patients showed a higher prevalence and severity of plaque progression after stenting; (2) the larger number of progressive lesions observed in the DM group distributed on LM, RCA and proximal LAD, and were characterized by more frequent non-stenotic segments and non-calcified plaques compared to non-DM groups; (3) diabetes was an independent risk factor for plaque progression in stented patients after the adjustment for confounding factors.

Despite the different clinical features of CAD patients, all exhibited multiple concomitant metabolic abnormalities, in which synergy affected the progression of coronary heart disease, increasing the risk of adverse cardiovascular events [18]. Previous studies confirmed that obesity or Mets aggravate the plaque burden and worsen the prognosis of CAD patients [19]. Statins reduce the plaque/lipid composition, increase plaque stability, and improve adverse cardiovascular events through lowering circulating lipid levels. Studies have shown that during a relatively short follow-up (~ 1 year), moderate doses of statins do not significantly change the plaque phenotype and increase calcified plaque volumes [20–22]. A possible explanation for this is the limited time window to exert an effect.

In this study, although a small number of patients did not receive optimal statin use in accordance with treatment guidelines after discharge, no significant differences in statin usage between DM and non-DM groups were observed. In addition, DM patients are susceptible to a higher prevalence of CAD and plaque progression, even when receiving lipid-lowering therapy [23, 24]. This implies that DM may be an independent risk factor for plaque progression.

DM markedly impaired myocardial microvascular perfusion which is regarded a clinically significant predictive marker of plaque progression in CAD [25–27]. Previous studies evaluating the use of serial CCTAs, verified that higher levels of plaque progression occurred in DM patients [4, 5]. Similar results were obtained in this study, which included elective PCI treated patients. Insulin resistance causes early endothelial dysfunction by decreasing eNOS activity and nitric oxide production. Monocyte/macrophage activation leads to enhanced inflammation in DM patients, which impairs endothelial functions and limits repair of the endothelium [28, 29]. These effects enhanced the severity of pre-existing atheromatous plaques and increased the prevalence of newly developed plaques in non-stenotic segments. Thus, strict glycemic control and intensive DM management should be prioritized to prevent CAD progression and secondary adverse cardiovascular events in DM patients.

Previous studies investigated the distribution of atherosclerosis between DM and non-DM groups, suggesting that proximal segments, particularly proximal LAD were more susceptible to plaques in DM patients [30]. Our data indicated that more progressive lesions in stented patients with DM were located in LM, RCA and the proximal LAD compared to patients without DM. Possible explanations for these observations are wall shear stress (WSS) in different parts of the coronary artery tree. It is recognized that low WSS is independently associated with an increased plaque burden and adverse plaque characteristics at follow-up [31–33]. WSS at the proximal segments, such as LM and proximal LAD, may be lower than those distal to the vessel. RCA is more likely to possess a lower WSS due to its relatively larger diameter.

In addition to WSS and compared to non-DM patients, circulating tissue factor procoagulants make DM patients susceptible to hypercoagulation [34]. Plasma viscosity, as an additional determinant of endothelial function and the maintenance of normal vascular resistance, can affect atheromatous plaque progression when increased. Hypercoagulability decreased the WSS so that the coronary artery trees in DM patients were exposed to higher levels of plaque progression compared to non-DM patients. LM and proximal LAD are located at the ostial of the vessels and when significant obstruction occurs, an insufficient blood supply to the downstream vessels and more extensive myocardial damage results. Further studies are now required to establish the exact relationship between coronary arrangements and plaque progression.

An additional finding was that, compared to non-DM patients, non-calcified plaques in DM patients showed higher rates of plaque progression. The quantification of non-calcified plaques can improve the prognostic value of CCTA to predict future cardiovascular events and is regarded as an independent predictor of developing acute coronary syndrome (ACS) [35, 36]. As the progression of non-calcified plaques in DM patients are more common after stenting than non-DM patients, they may be more at risk to ACS. Regular re-examination and evaluation of the plaque changes in the non-stent segments of DM individuals should be given attention.

Baseline disease increased the risk of plaque progression [37, 38]. Based on our data, the same correlation did not exist between baseline SSS or SIS and plaque progression. This may due to the study population being comprised of CAD patients who required PCI based on clinical decision making, which differs to the general population. An interesting discovery was that chest symptoms at baseline CCTA could effectively predict plaque progression. Chest pain was closely related to coronary plaque burden and long-term poor prognosis, facilitating the risk stratification of patients combined with cardiovascular risk factors [39, 40]. To-date, discussions on whether baseline chest symptoms affect plaque progression and prognosis in stent implanted patients are limited. Due to the single-center and retrospective nature of this study, we were unable to draw definitive conclusions. Further prospective, multi-center studies are required to identify the impact of baseline chest symptoms on CAD progression and prognosis in PCI treated patients, and to evaluate whether patients with chest pain require more aggressive therapy.

The present study had some limitations. First, the results were based on a small sample size and the inclusion of a larger study sample would strengthen the statistical analyses. Secondly, the study was retrospective and single-centered, leading to potential selection bias. The results remain to be verified by prospective and multicenter studies. Thirdly, we did not systematically correlate our findings on CCTA with angiography for luminal stenosis assessments, as the high-diagnostic accuracy of CCTA for the assessment of coronary atherosclerosis (which is comparable to angiography) is widely accepted. Finally, we recorded baseline chest pain, which is a relatively subjective indicator. Two extremes require assessments based on chest symptoms alone, to minimize subjective errors.

## Conclusions

DM patients have a higher prevalence and severity of plaque progression of non-stented segments after PCI. Compared to non-DM individuals, more progressive lesions were located in LM, proximal LAD and the non-stenotic segments of DM-patients, which were characterized as non-calcified plaques. These findings suggest that initially non-obstructive disease may over time progress to significant stenosis, resulting in non-target-lesion PCI, particularly in DM patients. Physicians therefore should pay attention to non-stent segments and the management of non-stent segment plaque progression.

## Data Availability

The datasets used and analyzed during the current study are available from the corresponding author on reasonable request.

## Abbreviations

DM: diabetes mellitus
CAD: coronary artery disease
CCTA: coronary computed tomography angiography
PCI: percutaneous coronary intervention
SIS: segment involvement scores
SSS: segment stenosis scores
DES: drug eluting stents
DES: drug eluting stents
ΔSSS: segment stenosis score change
MACE: main adverse cardiovascular events
LM: left main
RCA: right coronary artery
LAD: left anterior descending
LCx: left circumflex
CVD: cardiovascular disease
ACS: acute coronary syndrome
